# Phosphorylation and compactness of neurofilaments in multiple sclerosis: Indicators of axonal pathology^☆^

**DOI:** 10.1016/j.expneurol.2008.06.008

**Published:** 2008-10

**Authors:** Axel Petzold, Djordje Gveric, Mike Groves, Klaus Schmierer, Donna Grant, Miles Chapman, Geoffrey Keir, Louise Cuzner, Edward J. Thompson

**Affiliations:** a Department of Neuroinflammation, Institute of Neurology, Queen Square, London, WC1N 3BG, UK; b Department of Pathology, Institute of Neurology, Queen Square, London, WC1N 3BG, UK

**Keywords:** AL, acute lesion, ALP, alkaline phosphatase, BSP, brain-specific proteins, CL, chronic lesion, CNS, central nervous system, CTRL, control group, EDSS, expanded disability status scale, ELISA, enzyme linked immunoabsorbant assay, EM, electron microscopy, GM, gray matter, IQR, interquartile range, MS, multiple sclerosis, NAWM, normal-appearing white matter, Nf, neurofilament, NfH, neurofilament heavy chain, NfL, neurofilament light chain, PP, primary progressive, RR, relapsing remitting, SAL, subacute lesion, SAPK, stress stress-activated protein kinase, SP, secondary progressive, WM, white matter, Neurofilament phosphoforms, Multiple sclerosis, Axonal injury

## Abstract

**Aims:**

Axonal pathology extends to the axonal cytoarchitecture leaving its signature on axoskeletal proteins. This study investigated whether neurofilament (NfH) phosphorylation would relate to the dynamics of axonal pathology in multiple sclerosis (MS).

**Methods:**

NfH phosphoforms (SMI32, SMI34, SMI35) were quantified by ELISA from microdissected samples of control and MS brain and spinal cord. Individual axons were analysed by electron microscopy, densitometrically and morphologically in adjacent tissue sections. Experiments were carried out pre- and post enzymatic dephosphorylation.

**Results:**

In control tissue a rostro-caudal gradient of NfH indicated an increase in axonal density from the brain gray matter towards the spinal cord. The highest levels of phosphorylated and hyperphosphorylated NfH were found in acute lesions of brain and spinal cord, in contrast to chronic lesions where levels were lower than in white matter, consistent with axonal loss. Dephosphorylated NfH was higher, but less densly packed in MS white matter axons compared to control tissue.

**Conclusions:**

The findings suggest that a less organised/compact axoskeleton or impaired axonal transport may represent an early sign of axonal pathology within the normal appearing white matter in MS. Subsequently a proportional increase of dephosphorylated NfH, aberrant phosphorylation and/or aggregation may occur whilst the protein is transported through the white matter towards the MS plaque, where hyperphosphorylated NfH dominates.

## Introduction

**Fig. 1:**
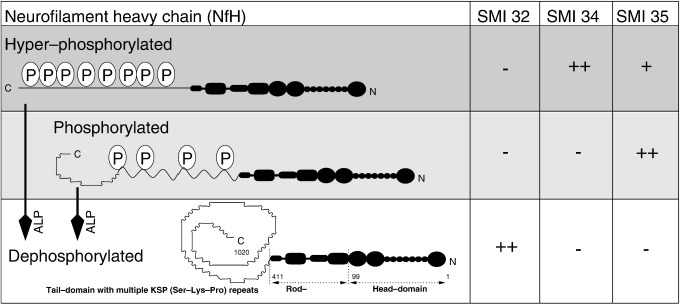
Conformational changes of the NfH protein due to phosphorylation expose and hide antibody-binding epitopes. Hyperphosphorylated NfH is recognised by the antibodies SMI34 and SMI35 and phosphorylated NfH by SMI35. Dephosphorylated NfH is strongly recognised by the antibody SMI32. ALP = alkaline phosphatase.

Multiple sclerosis (MS) is an inflammatory demyelinating disease in which axonal loss (Ferguson et al., 1997; Trapp and Peterson, 1998; Bitsch et al., 2000), thought to be the main pathological substrate for irreversible disability, is the dominant pathological feature of the degenerative phase of the disease (reviewed in (Lassmann et al., 2007; Bjartmar and Trapp, 2001; Esiri, 2007). In chronic MS patients about 50% of demyelinated spinal cord axons showed an abnormally fragmented axoplasm compared to about 4.8% of myelinated axons (Dutta et al., 2006). The finding was attributed to altered neurofilament (Nf) spacing. Neurofilaments are proteins specifically designed for post-translational modifications within the axonal compartment. Neurofilament phosphorylation increases Nf spacing and thereby axonal caliber (Grant and Pant, 2000; Lee and Cleveland, 1996; Petzold, 2005). The distinguishing feature of the Nf heavy chain (NfH) is its long tail with multiple KSP repeats acting as the main phosphorylation sites (see Fig. 1). Phosphorylation of the NfH KSP repeats is influenced by Ca^2+^ influx, glutamate, NGF and fibrin (Grant and Pant, 2000; Brownlees et al., 2000; Schwarzschild et al., 1999; Xia et al., 1996; Akassoglou et al., 2002). All of these mechanisms feature in the pathophysiology of MS. The pattern of NfH phosphorylation in staged lesions of MS and control brain tissue is not known.

In the present study we investigate the quantitative distribution of NfH phosphoforms in microdissected brain and spinal cord tissue with the aim of (1) outlining the topography of NfH phosphoforms in control tissue and (2) comparing NfH phosphoforms from control tissue with the lesion-specific NfH phosphoform distribution in MS tissue.

## Materials and methods

### Subjects

Patients with secondary MS according to the Poser criteria and age-matched controls were included in the study. All MS patients were classified as secondary progressive with significant disability (EDSS 9–10) (Gveric et al., 2001). Tissue was kindly provided by the Neuroresource Tissue Bank at the Institute of Neurology. We have previously published on this cohort with regard to glial biomarkers(Petzold et al., 2002) and the fibrinolytic system in brain tissue (Gveric et al., 2001; Gveric et al., 2003). Tissue sampling has been extended and spinal cord tissue was included to enable a more global and systematic assessment in the present study. In total, 77 snap-frozen tissue blocks (0.5–1 cm^3^) were analysed.

### Immunocytochemistry

For immunocytochemistry, 10 µm cryostat sections were immunoperoxidase-stained with antibodies directed against SMI32, SMI34 and SMI35 (Sternberger Monoclonals Inc., Lutherville, USA), GFAP, (Newcombe, 1986) 14E for oligodendrocytes and reactive astrocytes (Newcombe et al., 1992). Cryostat sections were fixed in methanol (− 20 °C, 10 min), incubated with primary antibody overnight (4 °C) and stained using a three-step peroxidase method (Gveric et al., 1999). Adjacent sections were incubated with 0 mU (control), 10 mU, 100 mU or 1000 mU alkaline phosphatase (Sigma, P4252) in Tris–HCl buffer (100 mM Tris, pH 8.1 with 1% Triton X-100), at room temperature for 18 h prior to immunostaining as described (Sternberger and Sternberger, 1983). Omission of primary antibodies and the application of affinity purified rabbit (Dako), goat (Sigma) and mouse IgG (Sigma) were used as controls at the same protein concentrations as the appropriate primary antibodies. Images were photgraphed on a Leitz DMRB microscope using a 1/125 exposure time on a Nikon Coolpix 995 camera (3.34 megapixels).

### Lesion classification

Unfixed post-mortem brain tissue was histologically classified into normal-appearing white matter (NAWM), acute lesions (AL), chronic lesions (CL) and gray matter (GM) from MS patients using previously published criteria (Newcombe and Cuzner, 1996). Adjacent pieces of each type of tissue were excised and homogenised as described (Petzold et al., 2003a,b). This study did not include remyelinated lesions or cortical demyelination.

### Electron microscopy

Standard protocols were used for electron microscopy (EM). In brief, post-mortem brain tissue samples were taken from the frontal periventricular white matter and fixed for 1 h in 4% glutaraldehyde in 0.1 M sodium cacodylate buffer (pH 7.4). The samples were then cut into 1 mm cubes, washed in PBS and processed into araldite CY 212 for EM. Ultrathin sections were cut from the surface of the tissue, stained with uranyl acetate and lead citrate, and examined at 80 kV.

### Image analysis

Images were analysed on a LINUX workstation using the GNU Image Manipulation Program (GIMP is distributed under the GNU General Public License and can be obtained free of charge from www.gimp.org). For densitometric analysis sections of individual axons were used. For each axon, the diameter was measured using the number of pixels as an arbitrary unit. Next, a histogram of the pixel intensity was obtained on a grey-scale ranging from 0 (black) to 255 (white). The mean pixel intensity (± SD), range and the absolute pixel number per axon were recorded. Because background intensity varied between slides, the mean background intensity was subtracted from the mean intensity for each axonal measurement. The estimated optical density (OD), was then expressed as the ratio of 1 to the mean pixel intensity as an arbitrary unit. Finally, the ratio of OD to axonal diameter was used in order to express the compactness of axonal NfH.

### Protein extraction

Snap-frozen blocks of brain tissue from MS and control cases of between 0.5 g and 1 g wet weight, were cut and re-suspended at 1:5 g/mL in Tris–HCl buffer (100 mM Tris, pH 8.1 with 1% Triton X-100). A protease inhibitor cocktail (Sigma, P 8340) was added in a dilution 1:100. Samples were homogenised on ice by sonication, triturated 3 times through 19 and 21 gauge needles and spun at 20,000 *g*. In order to de-lipidise the sample diisopropyl ether was added. After extensive mixing, the sample was spun at 20,000 *g*. The supernatant was covered by a myelin layer. A needle was put through the myelin layer and only the supernatant drawn up into a 1 mL syringe. The myelin layer and pellet were decanted. The supernatant was aliquotted into 2 portions.

### Dephosphorylation

It has been suggested that dephosphorylation uncovers epitopes particularly accessible to SMI32, which are otherwise hidden (Sternberger and Sternberger, 1983) (Fig. 1). Therefore one aliquot of the tissue homogenate was subjected to enzymatic dephosphorylation by using alkaline phosphatase (ALP, Sigma, P4252) which dephosphorylates Nf (Grant and Pant, 2000).

Barbitone buffer was selected for NfH incubation with ALP because it is inert to enzyme activity (Moss and Henderson, 1994, p. 831). The optimum pH for the different alkaline phosphatase isoforms varies from about 8.5 to 10.5, thus enabling direct incubation into the sample buffer (pH 8.9) (Petzold et al., 2003a,b).

### Western- and Immunoblotting

A tris-acetate gel (3–6%, NuPage) was used for Western- and immunoblotting. The gel was loaded with 5 μL of molecular weight markers (Invitrogen, Novex P/N57318 for the Coomassie stain and MagicMark XP P/N LC5602 for chemiluminescence). Thirty microlitre of sample were loaded per lane. The sample contained either purified NfH (12.5 μg/mL), purified NfH (12.5 μg/mL) together with ALP (50 μg/mL) or ALP alone (50 μg/mL). The gel was run for 1.5 h using TrisAcetate SDS running buffer (Novex, LA0041) at 150 V. The gel was cut in three parts: lanes 1–4 for Coomasie stain, lanes 5–7 for incubation with the SMI34 antibody and lanes 8–10 for incubation with the SMI32 antibody. For the immunoblot proteins were transferred onto nitrocellulose (Novex transfer buffer, NP00061, at 25 V for 2 h). The membranes were blocked in 5% skimmed milk (barbitone buffer) for 1 h. The membranes were washed and incubated in 0.1% skimmed milk (barbitone buffer) with monoclonal mouse anti-NfH antibodies (SMI34 1:1000, SMI32 1:1000) at 4 °C overnight. The membranes were washed 6 times for 10 min, incubated with HRP-labeled rabbit anti mouse (DAKO, P0260, 1:1000) and washed as before. The membranes were incubated with the chemiluminescence substrate (SuperSingal West Pico, Thermo Scientific, #34078) for 5 min. The dried membranes were visualised on a AlphaEase FluorChem SP CCD camera (20 s for SMI34 and 7 min for SMI32).

**Fig. 2:**
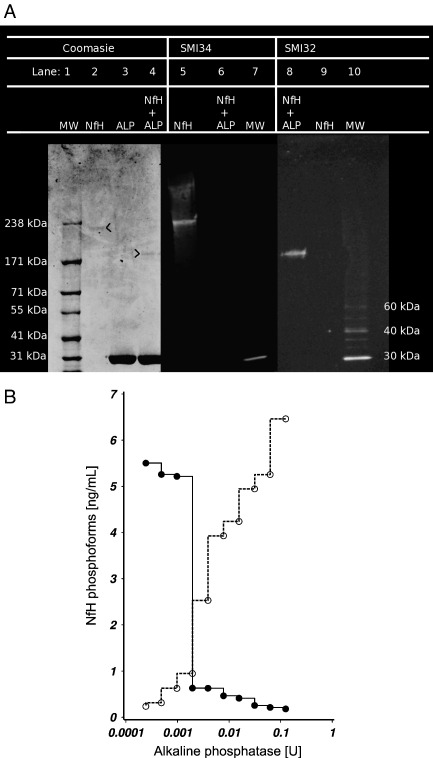
Enzymatic dephosphorylation of purified NfH with ALP. (A) The Western blot shows a band for phosphorylated NfH (lane 2) at ≈ 210 kDa and for ALP (31 kDa) dephosphorylated NfH (lane 4) at ≈ 190 kDa. The immunoblots demonstrate that SMI34 binds selectively to phosphorylated NfH (lane 5) and SMI32 selectively to ALP dephosphorylated NfH (lane 8). (B) Dephosphorylation of NfH with ALP is dose dependent. Incubation with ≈ 2 mU ALP at 37° for 2 h (pH 10.3) is needed to mask the phosphorylated epitopes recognised by the SMI34 (closed line) and unmask the non-phosphorylated epitopes recognised by SMI32 (dotted line).

The Coomassie stain shows a band at approximately 200 kDa for phosphorylated NfH (lane 2 in Fig. 2A). ALP gives a strong band at ≈ 31 kDa (lanes 3 and 4). ALP dephosphorylated NfH shows a band at ≈ 190 kDa (lane 4). The immunoblots show that SMI34 binds selectively to phosphorylated NfH (lane 5) and SMI32 to dephosphorylated NfH (lane 8). There is no cross-reactivity of either antibody with ALP.

### Assays

Levels of NfH phosphoforms were quantified using an in-house ELISA technique as described (Petzold et al., 2003a,b). All samples were analysed in duplicate and repeated if the error between duplicates exceeded 10%. The monoclonal antibodies SMI32, SMI34 and SMI35 were purchased from Sternberger Monoclonals Inc. (these antibodies are now supplied by Covance). Fig. 1 summarises the antibody binding properties.

As proof-of-principle HPLC-purified bovine NfH (Affiniti Research Products) was incubated with ALP at different concentrations ranging from 0.2 mU to 125 mU for 2 h at 37 °C. ALP denatures above 56 °C, therefore incubation for 10 min at 60 °C was used for stopping the enzymatic reaction. The untreated control samples were subjected to the same procedure and incubated with an equal volume of sample buffer. The effect enzymatic dephosphorylation has on covering the epitopes recognised by SMI34 and uncovering the epitopes recognised by SMI32 is shown in Fig. 2. Complete dephosphorylation of the phospho-epitope recognised by SMI34 was achieved following dephosphorylation with 2 mU ALP. However unmasking of the non-phosphorylated epitope recognised by SMI32 required a higher concentration of ALP with approximately 75 mU. For this reason the patients' samples were incubated with 100 mU of ALP for 18 h at room temperature. Fig. 2B demonstrates that the degree of dephosphorylation was sufficient to completely mask the epitope recognised by SMI34 (shown in the immunoblot in Fig. 2A) from the spinal cord homogenates. All aliquots were then stored at − 70 °C until further analysis.

Total protein was determined using the Bio-Rad Protein assay (Bio-Rad, Hemel Hempstead, UK).

### Data analysis

All statistical and data analyses were carried out using SAS (version 9.1, SAS Institute, Inc., Cary, North Carolina, USA). The 2-tailed non-parametric two-sample exact Wilcoxon rank–sum test was used for comparing two groups and a 2-tailed two-way unbalanced ANOVA (general linear model, GLM) for more than two groups. If significance was based on small numbers (*n* < 10), the results were also subjected to Fisher's exact test comparing proportions of samples above or below cut-off. The cut-off was defined as the 100% cumulative frequency of the subgroup with the lowest values. Trend analysis was done using Mantel–Haenzels *χ*^2^ test. Correlation analysis was performed using the Spearman Correlation Coefficient followed by the Bonferroni correction in case of multiple analyses. A *p*-value of < 0.05 was accepted as significant if shown on the discrete (GLM and post-hoc) and the categorical level (Fisher's exact test). The *p*-values for the GLM post-hoc analysis are given in the Tables and for Fisher's exact test in the Figures.

## Results

The patients' characteristics are summarised in Table 1. There were no significant differences in the age distribution or the post-mortem interval, but there were more females in the MS group than in the control group (*p* < 0.05).

There was no correlation for any of the NfH phosphoforms with either the post-mortem interval, the age or the disease duration.

**Fig. 3:**
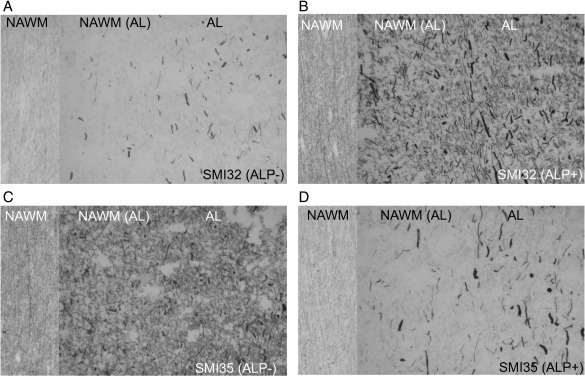
Immunocytochemistry for phosphorylated (SMI35) and dephosphorylated (SMI32) NfH in longitudinal sections along axonal trajectories from normal-appearing white matter [NAWM] far away from any lesion (left section in the slides), normal-appearing white matter adjacent to the acute lesion [NAWM (AL), central section in the slides] and the acute lesion [AL, right section of the slides]. The sections were incubated with (A, C) buffer only (0.1 M Tris–HCl pH 8.0) or (B, D) 100 mU ALP in buffer for 18 h at 37 °C before immunostaining with either SMI32 or SMI35 (× 10).

**Fig. 4:**
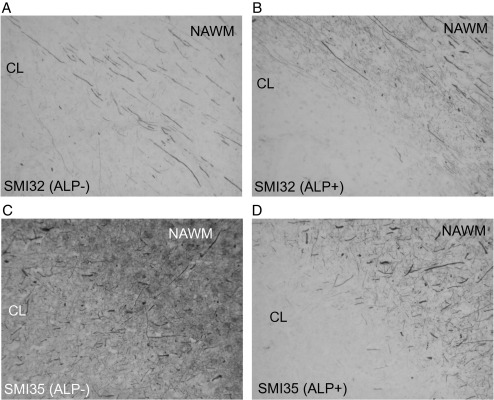
Immunocytochemistry of a chronic plaque featuring dephosphorylated NfH (A, B) and phosphorylated NfH (C, D). (A, C) show untreated and (B, D) sections (× 10).

The effect of dephosphorylation (see Fig. 1) is demonstrated by immunocytochemistry in Fig. 3. In untreated MS tissue mainly axonal end-bulbs stained for SMI32 (Fig. 3A). Most end-bulbs were observed in the AL, some in the NAWM adjacent to the lesion and only very few in NAWM distal to lesions. Treatment with ALP uncovered previously hidden epitopes (Sternberger and Sternberger, 1983) and revealed a much richer network of axons (Fig. 3B) involving the entire NAWM. In contrast, staining for SMI35 which recognises phosphorylated epitopes on NfH was clearly reduced in NAWM after dephosphorylation and to a lesser degree in AL (Figs. 3C, D). At the border of chronic lesions (CL), treatment with ALP did reduce staining for SMI35 to a lesser extent (Figs. 4C, D), giving the impression that phosphorylated NfH might “pile up” (Fig. 4D). As before, enzymatic dephosphorylation increased the staining for non-phosphorylated NfH (Figs. 4A, B), again giving the impression that it may “pile up” at the border of the CL (Fig. 4B).

As expected from the immunocytochemical data, treatment with ALP almost abolished the levels of phosphorylated and hyperphosphorylated NfH in the ELISA (data not shown) and considerably increased the levels of dephosphorylated NfH. For this reason further analysis will only consider data from untreated samples for hyper-/phosphorylated NfH and ALP treated samples for data on dephosphorylated NfH (see Table 2).

### Topography of NfH phosphoforms in control tissue

#### NfH phosphoform distribution

**Fig. 5:**
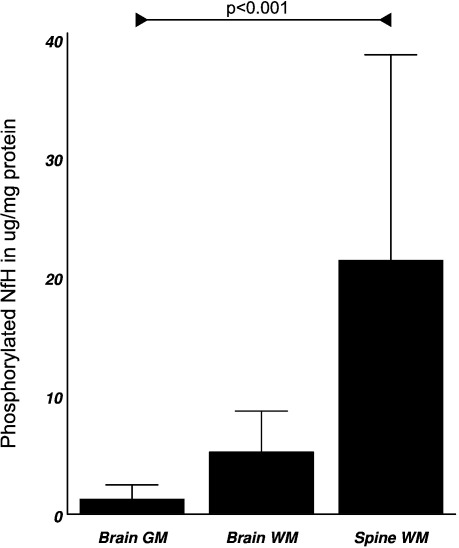
Rostro-caudal gradient of phosphorylated NfH in control tissue homogenate (mean ± SD). There was a significant trend for increasing levels of phosphorylated NfH from brain gray matter to brain white matter to spinal cord white matter (MH *χ*^2^ = 12.37, *p* < 0.001).

In control tissue we observed a rostro-caudal gradient of phosphorylated (Fig. 5 and Table 2A) and dephosphorylated NfH (Table 2B) with increasing concentrations from brain gray matter, to brain white matter, to spinal cord white matter. This trend was significant for phosphorylated NfH (M–H *χ*^2^ = 12.37, *p* < 0.01, Table 2A) and dephosphorylated NfH (MH *χ*^2^ = 7.4, *p* < 0.01, Table 2B).

Overall, the rostro-caudal gradient was most marked for phosphorylated NfH in control tissue (*F*_2*,*16_ = 5.53, *p* < 0.01) and to a lesser degree for dephosphorylated NfH (*F*_2*,*16_ = 4.17, *p* < 0.05). The phosphorylated NfH levels were about 3 to 15-fold higher in control spinal white matter compared to control brain gray matter or control brain white matter (*p* < 0.05, *p* < 0.01, respectively). Thus the percentage of phosphorylated NfH to the total soluble protein corresponds to 0.04% in the grey matter, 0.54% in the white matter and 1.71% in the spinal cord.

The dephosphorylated NfH levels were about 5-fold higher in control spinal cord white matter compared to brain gray matter (*p* < 0.05) or brain white matter (*p* < 0.05, Table 2B).

For hyperphosphorylated NfH the bulk of the protein was found in the control brain white matter (*F*_2*,*16_ = 6.79, *p* < 0.01) with levels being about 17-fold above levels in control brain gray matter (*p* < 0.01) and about 10-fold higher than levels of spinal cord hyperphosphorylated NfH (*p* < 0.01, Table 2A).

### Distribution of NfH phosphoforms in MS

#### Brain gray matter

There were no significant differences between controls and MS patients for any of the NfH phosphoforms (Table 2A and B).

#### Brain white matter

**Fig. 6:**
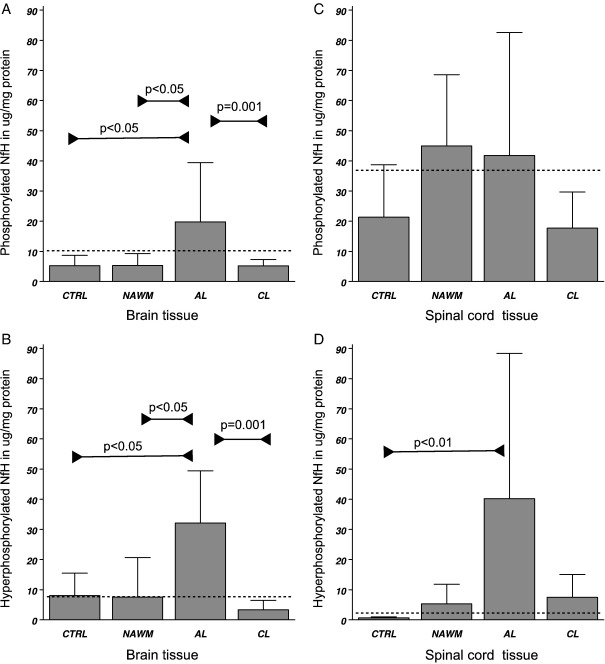
Tissue homogenate levels of (A) phosphorylated NfH and (B) hyperphosphorylated NfH in the brain white matter, (C) phosphorylated and (D) hyperphosphorylated NfH in the spinal cord white matter. Levels are shown in μg/mg protein (mean ± SD). Levels of significance are indicated in the graph (Fisher's exact test) and the cut-off value is shown (dotted line).

**Fig. 7:**
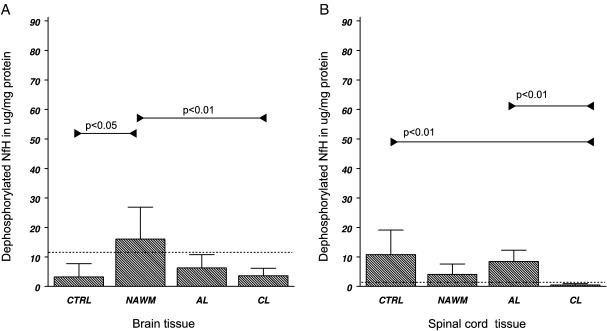
Tissue homogenate levels of dephosphorylated NfH in (A) the brain white matter and (B) the spinal cord white matter. Levels are shown in μg/mg protein (mean ± SD). Levels of significance are indicated in the graph (Fisher's exact test) and the cutoff value is shown (dotted line).

Significant differences in tissue levels were found in NAWM, AL and CL, for phosphorylated NfH (*F*_3*,*20_ = 3.34, *p* < 0.05), hyperphosphorylated NfH (*F*_3*,*20_ = 8.44, *p* < 0.001) and dephosphorylated NfH (*F*_3*,*20_ = 5.66, *p* < 0.01). The highest levels of phosphorylated and hyperphosphorylated NfH were found in AL (Table 2A). Significances were confirmed on a categorical level by Fisher's exact test (Figs. 6A and B). Dephosphorylated NfH was found to be highest in NAWM (Table 2B, Fig. 7A).

#### Spinal cord white matter

Significant differences in tissue levels were found for hyperphosphorylated NfH (*F*_3*,*24_ = 3.24, *p* < 0.05) and dephosphorylated NfH (*F*_3*,*24_ = 5.30, *p* < 0.01). Levels of hyperphosphorylated NfH were significantly higher in AL compared to control tissue (Table 2A and Fig. 6D). Dephosphorylated NfH was lowest in CL (Table 2B and Fig. 7B).

#### Differences between MS brain and spinal cord

The concentration of phosphorylated NfH was 2 to 8-fold higher in the spinal cord than in the brain for MS white matter (*p* < 0.01) and CL (*p* < 0.01). In contrast, dephosphorylated NfH was 7 to 13-fold higher in the brain compared to the spinal cord white matter (*p* < 0.05) and CL (*p* < 0.05). This was inverse to the pattern observed for dephosphorylated NfH in the control tissue (see above and Table 2B).

#### Image analysis

**Fig. 8:**
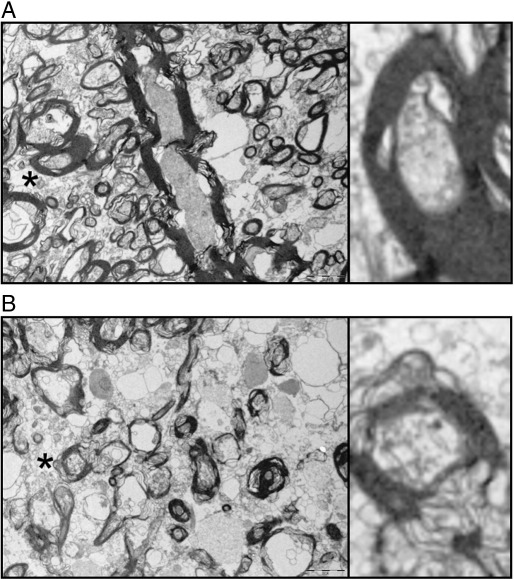
The ultrastructure of axons from the frontal periventricular white matter of (A) controls and (B) NAWM from MS patients is shown. The electron micrographs of several myelinated and unmyelinated axons illustrate the more fragmented ultrastructure of axons in NAWM. Additionally the density of the axonal cytoskeleton is less compact in NAWM axons. This is best appreciated in the inlay which shows an enlarged copy of two myelinated and approximately equally sized axons (indicated by a star and rotated by 90° for better visibility). Scale bars 2 µm.

The electron micrograph of the axonal ultrastructure is shown in Fig. 8. The images demonstrate the more disorderly axonal cytoskeleton in NAWM if compared to control white matter. Additionally, the spacing between the intermediate filaments is larger, suggestive of reduced compactness of Nfs in NAWM.

Samples from the periventricular control tissue and NAWM of the frontal periventricular white matter were analysed. A median of 3016 (636–20849) pixels per axon were examined from slides stained for SMI32 with and without ALP treatment. The median axonal diameter was 5.8 (3.0–23.3) pixels. There was a Gaussian distribution for small axons (≤ 10 pixels) and a bimodal distribution for large axons (data not shown). The median axonal OD was 81.1 (4.9–283.4, arbitrary units). There was no significant difference in axonal size between control tissue and NAWM with a median diameter of 15 pixels for large and 5.8 pixels for small axons for either experiment.

Enzymatic dephosphorylation with ALP uniformly affected axonal NfH compactness. There was a significant difference in the OD between groups (*F*_3*,*156_ = 87.15, *p* < 0.0001). The post-hoc analysis showed that the OD was higher in ALP-treated slides comparing large axons of control tissue (OD = 160.17) with large axons in NAWM (OD = 118.74, *p* < 0.001). Also, the OD of small axons in control tissue (OD = 92.74) was higher than in NAWM (OD = 63.65, *p* < 0.01). The OD was consistently higher in all ALP-treated axons than in untreated axons (*p* < 0.001 for each comparison). This explains the increase of axonal compactness of NfH after treatment with ALP in control tissue and NAWM (*p* < 0.001, *p* = 0.001, respectively).

**Fig. 9:**
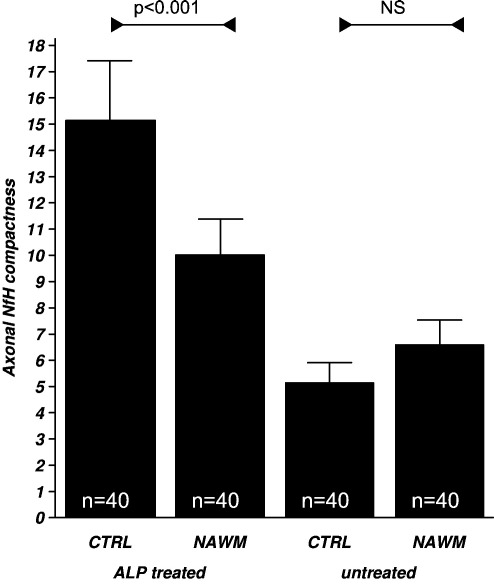
The compactness of axonal NfH is expressed as the ratio of OD to axonal diameter (mean ± SD) as explained in the methods. Compactness of NfH is significantly higher in ALP treated tissue homogenate from control compared to MS white matter. This effect was not observed in untreated tissue. Levels of significance are indicated (GLM, post-hoc analysis, see text).

Indeed, axonal compactness of NfH was significantly different between groups (*F*_3*,*156_ = 37.76, *p* < 0.001). The post-hoc analysis showed a higher degree of axonal compactness of NfH in ALP-treated control tissue compared to NAWM axons (*p* < 0.001, Fig. 9).

## Discussion

### NfH topography and compactness in control tissue

The significant trend for increasing concentrations of phosphorylated NfH (SMI 35, see Fig. 1) from the brain gray matter to brain white matter to spinal cord white matter suggests an increase in parenchymal axonal density. These results are consistent with recently reported findings in rodents (Shaw et al., 2005).

In view of the high levels of hyperphosphorylated NfH (SMI 34) in brain white matter, the finding of a rostro-caudal gradient for dephosphorylated NfH (SMI 32) needs to be interpreted on basis of the original work on enzymatic dephosphorylation by ALP (refer to Fig. 1). According to Sternberger *et al.* ALP either uncovers hidden epitopes by dephosphorylation, (Sternberger and Sternberger, 1983) causes conformational changes, or releases NfH from protein/organelle binding (i.e. binding to microtubules). It was also suggested that phosphorylation increases compactness and order in Nf structure (Sternberger and Sternberger, 1983). In contrast, the dephosphorylated forms of Nf are more easily transported along the microtubule tracks, and the degree of phosphorylation correlated inversely with the velocity of axonal transport (Watson et al., 1989).

The present findings suggest that the degree of NfH phosphorylation and compaction may increase from the brain gray matter to white matter and further to the long tract axons of the spinal cord. We believe this is of biological relevance as will be seen below.

### NfH levels are reduced in chronic lesions

Both for brain and spinal cord tissue the lowest levels of dephosphorylated NfH were found in CL. This was consistent with the immunocytochemical observation that staining for dephosphorylated NfH was reduced in CL and its border region. These findings are in line with the observation of axonal loss in CL (Ferguson et al., 1997; Trapp and Peterson, 1998; Lovas et al., 2000). The more marked reduction of dephosphorylated NfH in CL of the spinal cord compared to the brain, which in fact is the inverse pattern to what we observed in control tissue, suggests some heterogeneity of the pathological process affecting brain and spinal cord axons.

Phosphorylated NfH appeared to “pile up” at the border of the CL (Fig. 4A–D). This contamination with phosphorylated NfH from the border region probably explains why, in contrast to dephosphorylated NfH, no significant difference was found compared to NAWM.

### NfH phosphorylation is abnormal in NAWM

The finding of enzymatically dephosphorylated NfH levels being approximately 7-fold higher in NAWM compared to control tissue, is corroborated by our immunocytochemical results. This difference is better appreciated from ELISA results, probably owing to a more effective exposure of epitopes during protein extraction if compared to antibody incubation of the intact tissue slide. This probably indicates an increase of aberrantly phosphorylated and/or aggregated NfH in brain white matter axons of these patients with secondary progressive MS. Not only is there an absolute increase of dephosphorylated NfH but the electron micrographs and image analysis also suggest that it is more diffusely distributed within the NAWM axons. Compared to control tissue axonal compactness of NfH was significantly reduced in NAWM. Importantly, this was independent of axonal diameter. This finding extends previous observations, (Sternberger and Sternberger, 1983) and suggests post-translational alterations of the axoskeleton at early stages of axonal pathology. We note that because all PM tissue was from patients with secondary progressive MS it is not possible to say from our data whether changes in NfH phosphorylation are a feature at onset of the disease. In order to address this question future studies may require biopsy material from patients with a clinical isolated syndrome or early relapsing remmitting MS. Because the increase of axonal compactness of NfH was only observed after incubation with ALP, we suggest that epitopes were hidden and/or the protein was bound, i.e. to microtubules and that initially the epitopes were not accessible to the SMI32 antibody.

### NfH phosphorylation is increased in acute MS lesions

The highest levels of phosphorylated and hyperphosphorylated NfH were found in AL (Fig. 3). Immunocytochemistry results showed a high amount of staining for SMI34 and SMI35 in axonal end-bulbs and thick axons bordering the AL. This suggests a volume-effect of accumulated phosphorylated and hyperphosphorylated NfH in this region which is greater than the relative NfH scarcity in the centre of the plaque, but the possibility of an additional extracellular component also needs to be considered. The existence of extracellular, mainly aggregated and phosphorylated NfH is known in the dementia literature (reviewed in (Petzold, 2005). Extracellular NfH which is not bound to tangles might be disregarded as non-specific background and thus escape the immunocytochemical observation. Consistent with one (Bitsch et al., 2000) but in contrast to other immunocytochemical observations (Trapp and Peterson, 1998; Geurts et al., 2003; Pitt et al., 2000; Gilgun-Sherki et al., 2003; Werner et al., 2001) we were not able to demonstrate a quantitative difference for hypo-phosphorylated NfH levels between AL, SAL and CL (data not shown). This may in part be related to the susceptibility of non-phosphorylated Nf to proteolysis (Sternberger and Sternberger, 1983; Goldstein et al., 1987; Pant, 1988).

### Is axonal transport in spinal cord axons impaired in MS?

The degree of NfH phosphorylation along the rostro-caudal gradient was substantially increased in MS if compared to CTRL tissue. Because NfH phosphorylation and axonal transport are inversely correlated (Watson et al., 1989; Ackerley and Grierson, 2000) this suggests an impairment of the axonal transport machinery, particularly in the long tract axons of the MS spinal cord. Impaired axonal transport may be an important pathological feature to be studies in demyelinating disease because of the intriguing relationship with dying back neuropathy (Coleman and Perry, 2002). Dying back neuropathy affects the *proximal* axon, but does not necessarily require axonal transection and also occurs diffusely e.g. in the context of chemotherapy, mitochondrial dysfunction or axonal Ca-overload. In contrast Wallerian degeneration affects axonal compartments *distal* to the lesion (Vargas and Barres, 2007). In MS Wallerian degeneration it thought to be the main mechanism by which axons degenerate, (Ferguson et al., 1997; Trapp and Peterson, 1998; Bitsch et al., 2000) a hypothesis supported by brain imaging techniques investigating axonal tract integrity distal to the lesion site (Ciccarelli et al., 2003). In the CNS axonal degeneration following injury may be substantially slower than in the PNS and take up to several years (Vargas and Barres, 2007). This is thought to be related to slow clearance of myelin-associated inhibitors of axonal growth such as myelin-associated glycoprotein (MAG), NogoA, oligodendrocyte-myelin glycoprotein (OMgp), semaphorin 4D and ephrin B3 (Vargas and Barres, 2007). Unfortunately, our study was designed to use de-lipidised samples preventing us from further investigating this exciting hypothesis on the present dataset, but future studies may be informative. Because Wallerian degeneration is irreversible, but dying back neuropathy may potentially be prevented it would be extremely important to try separating these two processes in MS. If, as suggested by the present data, dying back neuropathy should be a consistent feature in the MS CNS tissue, then such knowledge could guide the development of future neuroprotective treatment strategies.

A caveat of this study is that spinal cord tissue was taken only from patients with SPMS and an EDSS of 9 to 10, likely to represent the “burnt-out” phase of the disease. On basis of the MRI data, one would have expected to find severe atrophy in these spinal cords (Losseff et al., 1996). The almost complete absence of dephosphorylated NfH in CL of the spinal cord also supports the idea that tissue containing predominantly “burnt-out” axons has been sampled. Indeed, in patients with primary and secondary progressive MS we found an increase of phosphorylated and hyperphosphorylated NfH in the cerebrospinal fluid over a 3-year period (Petzold et al., 2005). An interesting speculation is that spinal cord axons might differ from brain tissue axons, thus contributing to the more insidious and inexorable progress of disease once affected. One biological explanation for this could be that for “topographical reasons of function” the spinal cord has to be hostile to axonal sprouting by exploiting the growth-inhibiting properties of proteins as mentioned above, the inhibition of which may be a new neuroprotective strategy in EAE (Karnezis et al., 2004). A 20 kDa soluble Nogo-A fragment (an axonal growth inhibitor (Schwab, 2004) was recently found to be present in 96% of CSF samples from MS patients, but none of the control patients (Jurewicz et al., 2007).

### Conclusion

In summary our data provides evidence that the proportion of dephosphorylated NfH is increased in MS. Whilst NfH is transported through the NAWM towards the MS plaque it may become aberrantly phosphorylated and/or aggregate and probably slow axonal transport itself. As NfH reaches the border of the acute plaque and in the spinal cord it becomes hyperphosphorylated. Quantification of NfH phosphoforms may provide a valuable tool to investigate the enzymatic machinery involved in the dynamics of neuro-axonal degeneration.

## Figures and Tables

**Table 1:** Subject characteristics

Characteristic	Controls	Multiple sclerosis	Significance
Number	15	20	
Sex (female:male)	3 (20%): 12 (80%)	11 (55%): 9 (45%)	*p* < 0.05
Age (yrs)	65 (34–80)	59 (29–77)	N.S.
Post-mortem interval (hours)	21 (11–52)	15.5 (4–71)	N.S.
Disease duration (yrs)	N/A	20 (8–43)	

*Cause of death*			
Heart failure	6 (40%)	1 (5%)	
Myocardial infarction	4 (27%)	0 (0%)	
Bronchopneumonia	2 (13%)	14 (70%)	
Pulmonary embolism	1 (7%)	0 (0%)	
Sepsis	0 (0%)	3 (15%)	
Renal failure	1 (7%)	1 (5%)	
Liver carcinoma	1 (7%)	0 (0%)	
Bowel carcinoma	0 (0%)	1 (5%)	

All data are shown as number (%), median (range), N.S. = not significant.

**Table 2:** Brain gray matter, white matter and spinal cord white matter tissue homogenate levels of (A) phosphorylated/hyperphosphorylated NfH in tissue homogenate and (B) enzymatically dephosphorylated NfH

NfH per μg/mg protein	CTRL	MS	AL	CL	Significance
*(A)*					
Brain GM:					
Phosphorylated (median) (IQR, *n*)	1.15 (0.14–1.90, 6)	2.07 (1.63–3.05, 6)	–	–	NS
Hyperphosphorylated	0.41 (0.22–0.78, 6)	0.24 (0.17–0.58, 6)	–	–	NS
Brain WM:					
Phosphorylated	5.47 (2.05–6.87, 6)	5.15 (3.30–5.47, 6)	12.8 (9.68–16.78, 6)	5.52 (3.98–6.87, 6)	*p* < 0.05^a^
Hyperphosphorylated	6.82 (4.90–8.16, 6)	2.45 (0.76–6.73, 6)	28.7 (26.59–46.02, 6)	2.96 (0.67–5.26, 6)	*p* < 0.001^b^*p* = 0.001^c^
Spinal WM:					
Phosphorylated	17.17 (8.44–25.92, 7)	43.65 (17.69–72.57, 7)	19.69 (12.46–60.38, 7)	13.93 (13.94–33.38, 7)	NS
Hyperphosphorylated	0.73 (0.40–0.86, 7)	2.21 (0.50–8.73, 7)	5.03 (3.62–86.27, 7)	5.21 (1.46–8.58, 7)	*p* < 0.01^d^

*(B)*					
Brain GM:					
Dephosphorylated	1.37 (0.62–2.64, 6)	1.34 (1.31–3.12, 5)	–	–	NS
Brain WM:					
Dephosphorylated	2.04 (0.31–3.05, 6)	15.29 (12.38–21.37, 6)	7.22 (2.45–10.12, 6)	3.73 (2.39–4.87, 6)	*p* < 0.01^e^*p* < 0.05^f^
Spinal WM:					
Dephosphorylated	10.46 (1.21–18.55, 7)	2.07 (1.42–5.40, 7)	9.12 (4.05–12.22, 7)	0.27 (0.15–1.08, 7)	*p* < 0.01^g^

The median value (IQR, *n*) are shown for normal control white matter (CTRL), normal-appearing white/gray matter (MS), acute lesions (AL) and chronic lesions (CL). The *p*-values are given if the GLM allowed for post-hoc analysis, NS = not significant.

a AL versus CTRL, AL versus MS (NAWM) and AL versus CL.

b AL versus CTRL.

c AL versus MS (NAWM) and AL versus CL.

d AL versus CTRL.

e MS (NAWM) versus CTRL and MS (NAWM) versus CL.

f MS(NAWM) versus CTRL.

g CL versus CTRL and AL versus CL.
